# Emerging trends in the nanomedicine applications of functionalized magnetic nanoparticles as novel therapies for acute and chronic diseases

**DOI:** 10.1186/s12951-022-01595-3

**Published:** 2022-08-31

**Authors:** Sabyasachi Dash, Tuhin Das, Paritosh Patel, Pritam Kumar Panda, Mrutyunjay Suar, Suresh K. Verma

**Affiliations:** 1 Weill Cornell Medicine, Medical College of Cornell University, New York, NY 10065 USA; 2 School of Biotechnology, KIIT, Bhubaneswar, Odisha 751024 India; 3 RayBiotech, Inc, 3607 Parkway Lane, Peachtree Corners, GA 30092 USA; 4 Memorial Sloan Kettering Cancer Center (MSKCC), New York, NY USA; 5 Department of Physics and Astronomy, Uppsala University, 75120 Uppsala, Sweden; 6 Technology and Business Incubator, KIIT, Bhubaneswar, Odisha 751024 India

**Keywords:** Magnetic nanoparticles, Si-RNA therapeutics, Vascular diseases, Neurological disorders, Nanomedicine

## Abstract

High-quality point-of-care is critical for timely decision of disease diagnosis and healthcare management. In this regard, biosensors have revolutionized the field of rapid testing and screening, however, are confounded by several technical challenges including material cost, half-life, stability, site-specific targeting, analytes specificity, and detection sensitivity that affect the overall diagnostic potential and therapeutic profile. Despite their advances in point-of-care testing, very few classical biosensors have proven effective and commercially viable in situations of healthcare emergency including the recent COVID-19 pandemic. To overcome these challenges functionalized magnetic nanoparticles (MNPs) have emerged as key players in advancing the biomedical and healthcare sector with promising applications during the ongoing healthcare crises. This critical review focus on understanding recent developments in theranostic applications of functionalized magnetic nanoparticles (MNPs). Given the profound global economic and health burden, we discuss the therapeutic impact of functionalized MNPs in acute and chronic diseases like small RNA therapeutics, vascular diseases, neurological disorders, and cancer, as well as for COVID-19 testing. Lastly, we culminate with a futuristic perspective on the scope of this field and provide an insight into the emerging opportunities whose impact is anticipated to disrupt the healthcare industry.

**Graphical Abstract:**

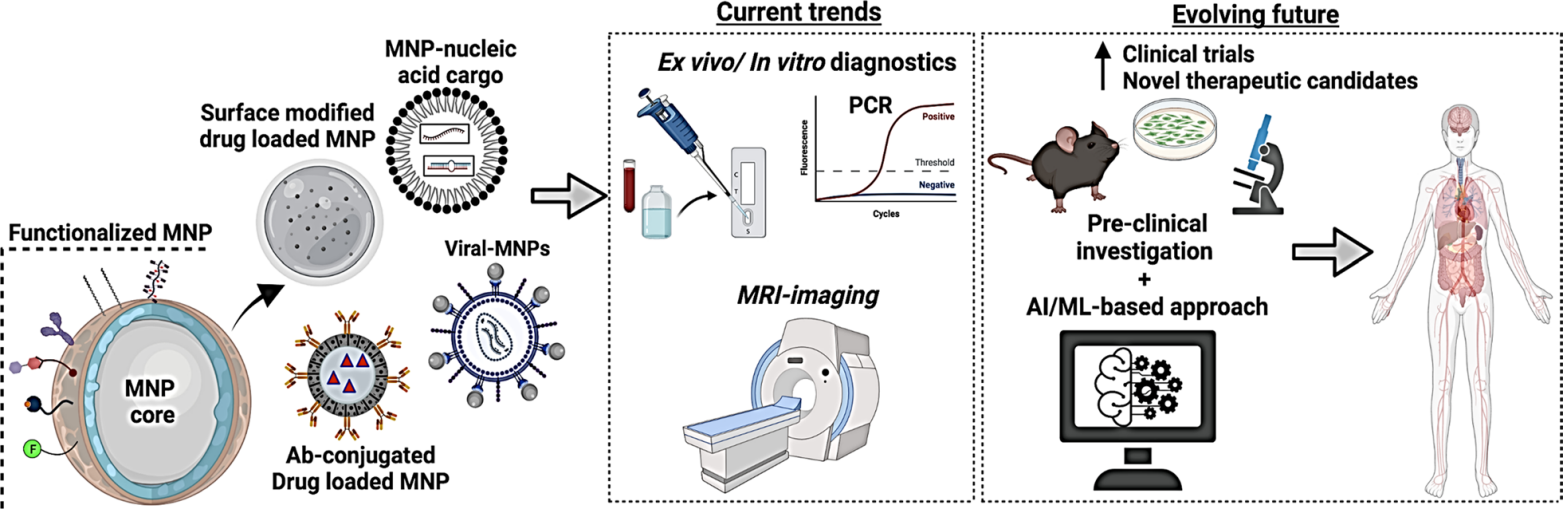

## Introduction

Magnetic nanoparticles (MNPs) are promising alternatives to fluorescently labeled biosensors. Structurally such particles consist of two major components—a magnetic material, (e.g. iron, nickel and cobalt), and a chemical component with analytical functionality [1]. Compared to the classical fluorescent, optical-based biosensors and magnetic detection harbors key advantages of low background with high signal and detection sensitivity [2–5]. With such functional versatility, magnetic nanoparticles have garnered tremendous attention in biomedical sciences with versatile applications in nanomaterial-based catalyst development, tissue specific targeting, microfluidics, pathogen detection, magnetic resonance imaging, magnetic particle imaging, environmental remediation, waste water remediation, optical filters, magnetic cooling and cationic sensors [6–12].

### Magnetic nanoparticle functionalization and synthesis

**Fig. 1:**
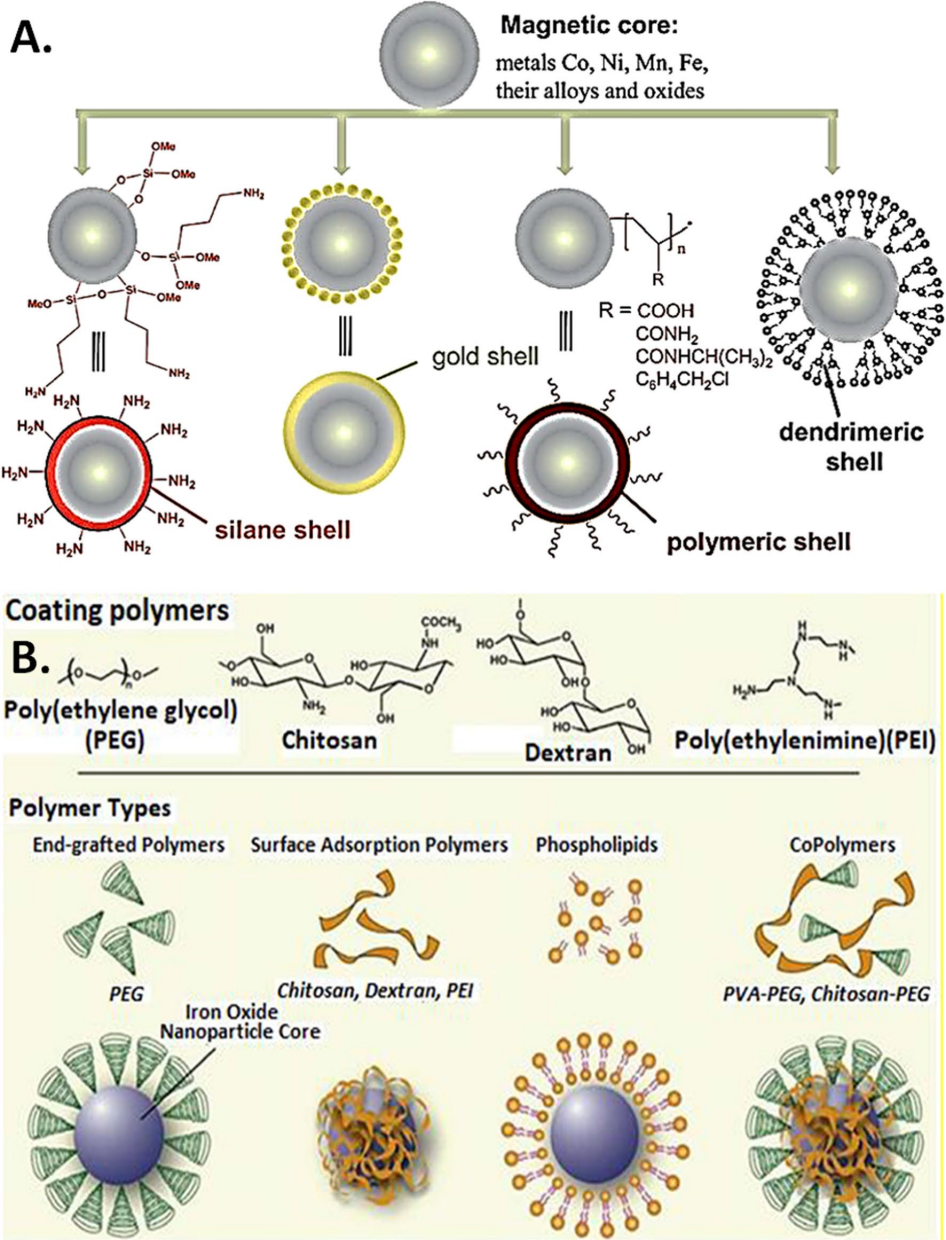
Functionalization types and features of core magnetic nanoparticles. **A** Illustration of magnetic nanoparticles prepared with various shells. (Reproduced with permission from Wilczewska et al. [34]). **B** Illustration for the assembly of various MNP surface functionalization methods. SPION surface coating is achieved via several methods including in situ coating, surface adsorption and end grafting. (Reproduced with permission from Veiseh et al. [18])

**Fig. 2:**
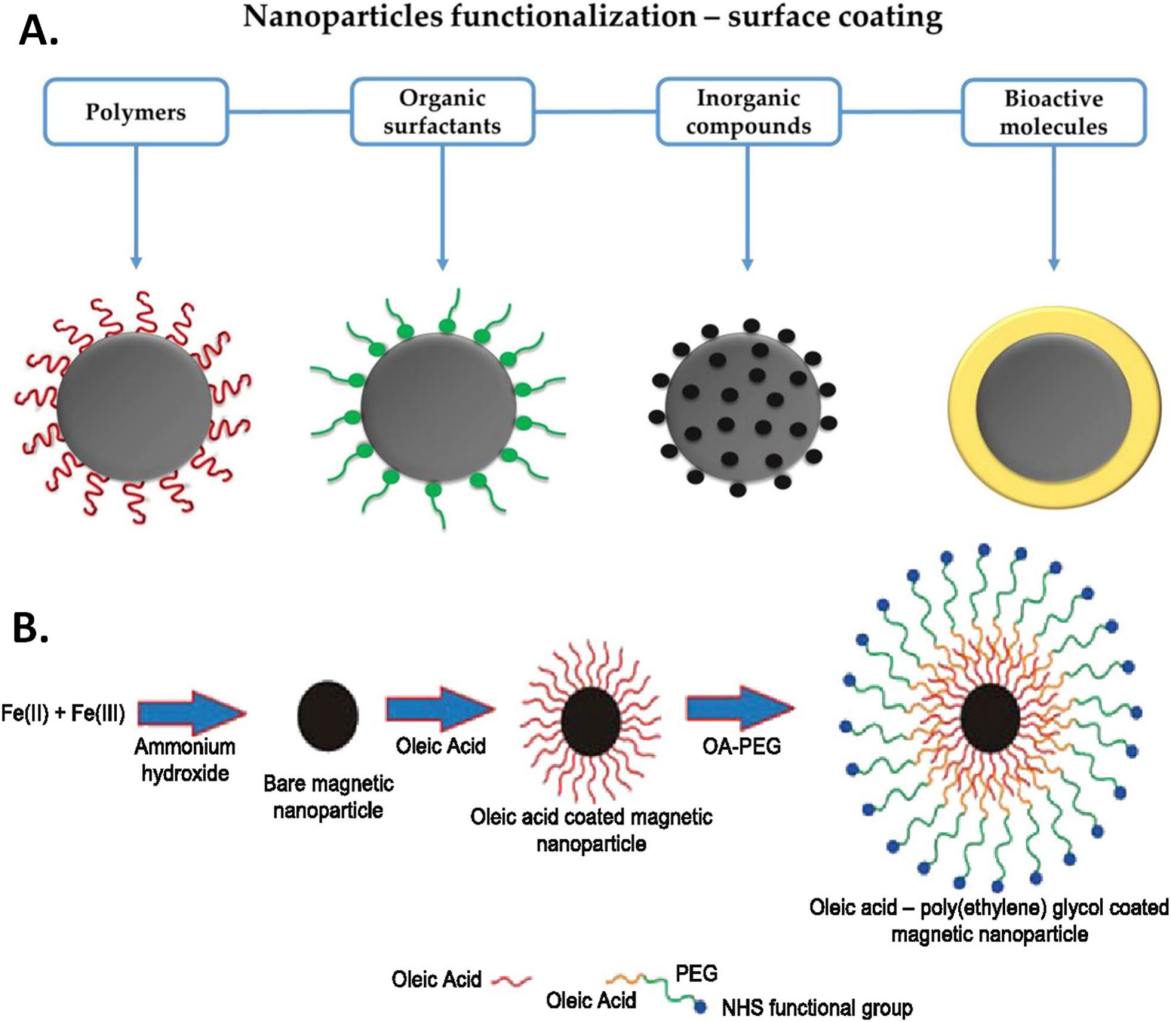
Surface functionalization of core magnetic nanoparticles. **A** Representative coating types used in iron-oxide core MNP functionalization (IONPs). Grey circles represent the core of IONPs. (Reproduced with permission from Arias et al. [35]). **B** Schematic for PEG-based MNP surface functionalization. Iron (Ferric hydroxide) based core was first surface coated with oleic acid (OA) and then functionalized with poly ethylene glycol (PEG) containing the *N*-hydroxysuccinimide (NHS) functional group for increased stability and drug binding. (Reproduced with permission from Yallapu et al. [36])

Several elegant methods are employed to synthesize MNPs such as, micro emulsion, thermal decomposition, co-precipitation, solvo-thermal, wave-based (sound, microwave), chemical vapor deposition, and combustion [13, 14]. Based on the application, the designing strategy involves several processes, such as (a) size-based synthesis, (b) identification of biocompatible linkers, ligands, (c) size vs. magnetism customization, and (d) identification of an optimal magnetic detection technique for functional validation [15]. The current technology allows the synthesis of magnetic nanoparticles selectively within a size range from the nanometer (nm) to the near micrometer (μm). For instance, when a magnetic nanoparticle is designed for in vivo applications, the preferred size is limited to ~ 100 nm for efficient endocytosis into the tissue/cellular microenvironment [13, 14, 16–18]. On the other hand, for biosensing applications a larger than 100 nm size is often preferred which features stronger signals owing to size-magnetism proportionality [14, 16–18]. During MNP functionalization, efficient coating of the magnetic core is ensured to preserve its surface functionalization (i.e. affinity towards target molecules) [18–21]. In general, surface coating of MNPs consists of two major approaches. In one approach, the synthesized MNPs are coated with organic/inorganic polymers (e.g., silica, carbon) while the other approach involves the in-situ coating by silica composites [22]. Derivatives of dextran, chitosan, polyethylene glycol (PEG), polyvinyl alcohol (PVA), oleic acid, polyoxamers, polyamines, which can be linked by chemical reagents or cross-linkers on the surface of MNP core (e.g., cobalt, nickel, manganese, iron oxide) are some of the most common coating materials, as depicted in Figs. 1 and 2. Given the importance of high-quality detection of functionalized MNPs, several widely employed magnetic detection techniques are currently in use including, spintronic sensors (magnetoresistance-based), and planar hall effect sensors, superconducting quantum interference devices (SQUIDs), atomic magnetometers, nuclear magnetic resonance (NMR) [23–27]. Nevertheless, optimizing magnetic nanoparticles for specific biological and interdisciplinary applications with appropriate detection methods, and precise sensitivity and specificity remains a key challenge.

The synthesis of magnetic nanoparticles is complex and demands attention on factors such as, (a) magnetic moment; a basic physical property of magnetic materials associated with the intrinsic spin angular momentum of the electrons; (b) susceptibility; one of the essential parameters for bulk magnetic materials comprising of the composition, crystallographic structure, vacancies and defects, which together determine the magnetic property; (c) saturation; defined as the maximum magnetization for a given magnetic material, which primarily depends upon the number of magnetic dipole, and finally the (d) biocompatibility; a critical property which ensures a compatibility of magnetic elements with the biological systems without losing its magnetism and sensing abilities [28–30]. Given the critical nature of biocompatibility, iron (Fe)-oxide based magnetic nanoparticles are more commonly used over others [28, 31]. However, experimental applications reveal a toxic profile of iron-oxide magnetic nanoparticles due to activation of reactive oxygen species (ROS), which are caused due to non-specific chemical reactivity whereas, for biosensing applications, MNPs with larger magnetic moments are preferred due to a stronger magnetic field that improves the sensitivity for detection/sensing [32, 33].

## Application of functionalized MNPs in pre-clinical and clinical research

**Fig. 3:**
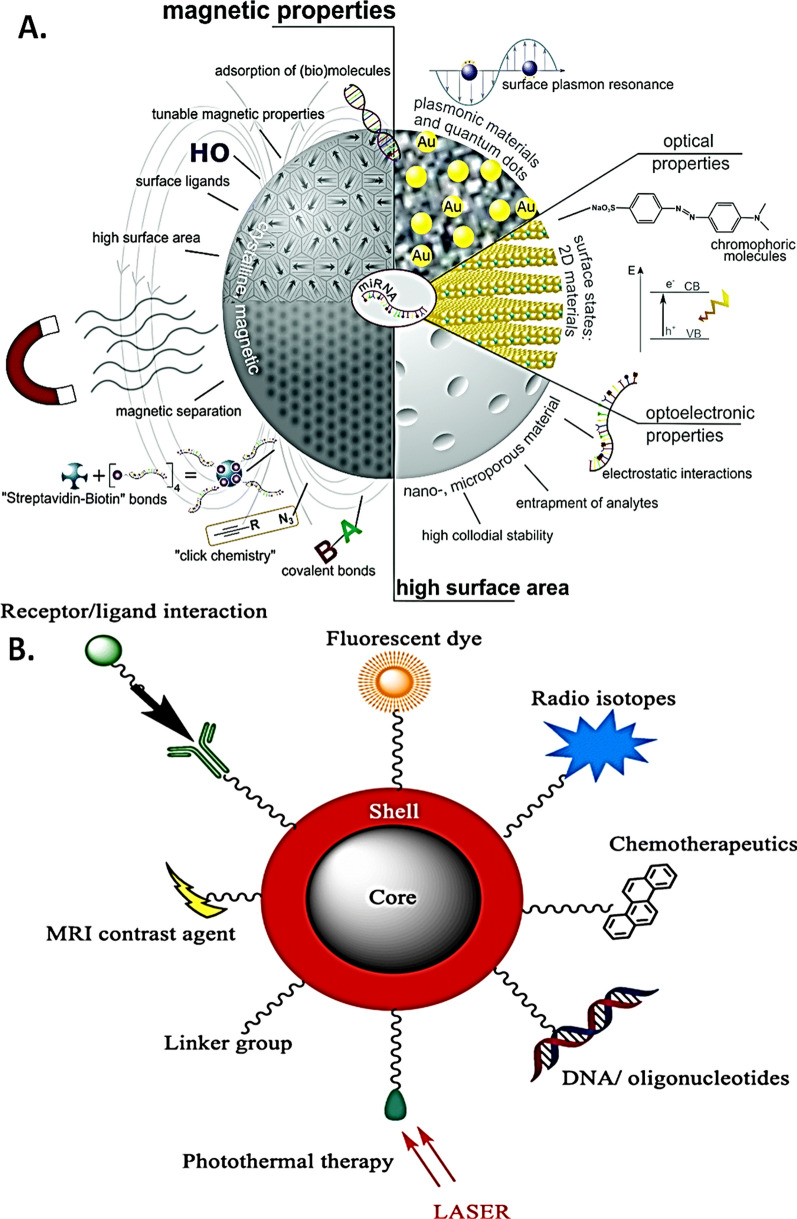
The many emerging applications of functionalized MNPs. **A** Schematic showing various nanomaterial’s including functionalized MNPs in sensing and detection of miRNAs. (Reproduced with permission from Gessner et al. [42]). **B** Illustration of analytics and biomedical applications of core shell functionalized MNPs. (Reproduced with permission from Anderson et al. [43])

Functionalized MNPs feature a high *surface-to-volume* ratio and can be easily manipulated in the presence of external magnetic fields with low background signals in biological samples [37]. Nevertheless, recent years have witnessed expansive applications of magnetic particles due to the fast-growing nanotechnology and attractive funding opportunities across both academia and industry. For instance, in the domain of point-of-care testing and biosensing, functionalized MNPs have been applied for mixing, capturing, enriching, and labeling of analytes [12, 38]. In principle, under a given magnetic field, dipole–dipole forces facilitate the formation of magnetic superstructures that allows the formation of one-dimensional nanostructured. Such assemblies exhibit a high surface-to-volume ratio, which allows their functionalization for efficient and stable target capture, sorting, isolation and enrichment (Fig. 3A). For specific capture events, bio recognition elements, such as antibodies and DNA probes, are employed to functionalize the surface of magnetic nanoparticles as illustrated in Fig. 3B [11, 39–41].

**Fig. 4:**
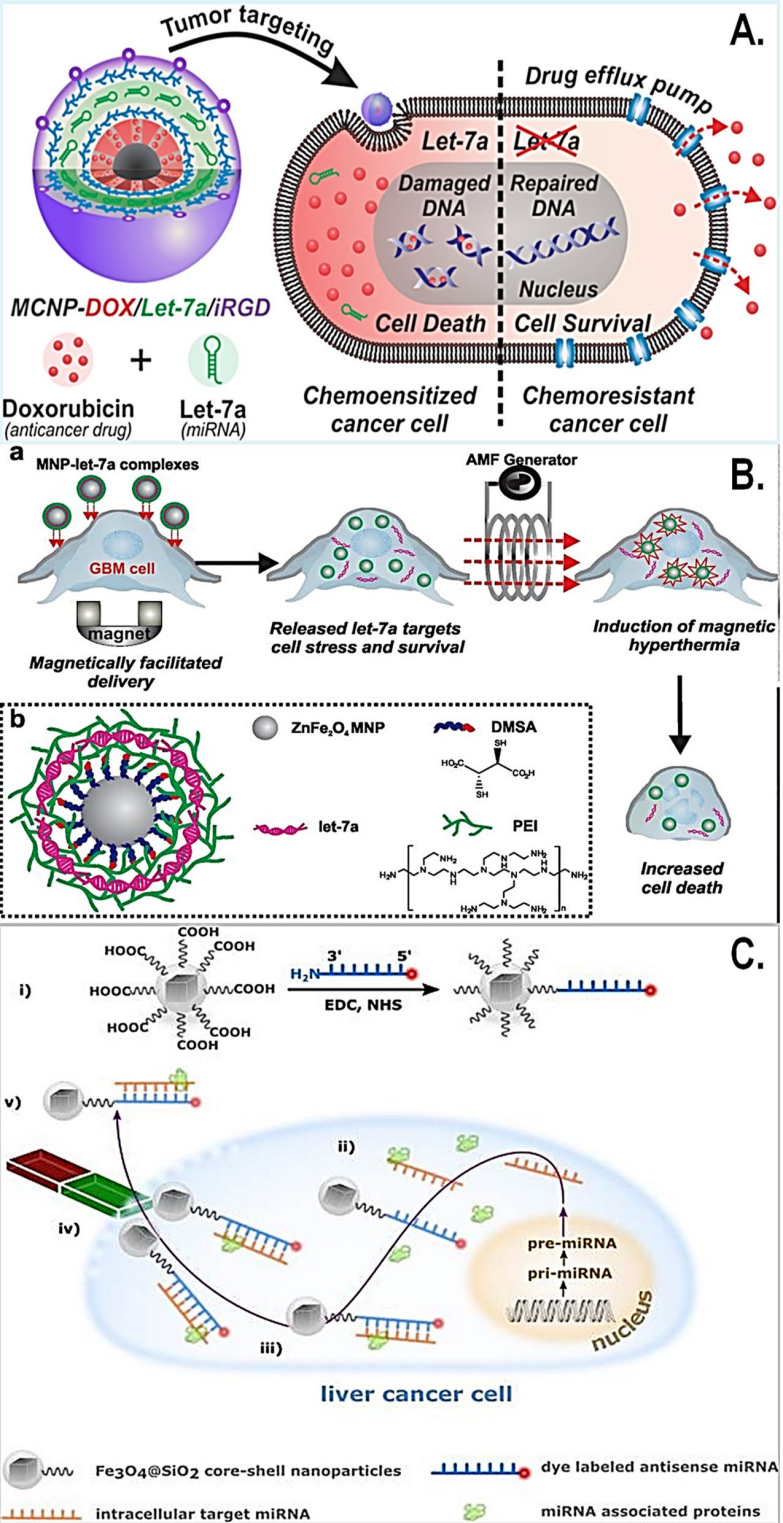
The many emerging applications of functionalized MNPs. **A** Application of a multifunctional magnetic core–shell nanoparticle (MCNP) constituting a magnetic zinc-doped iron oxide (ZnFe_2_O_4_) core nanoparticle surface modified with silica shell, for the dual delivery of miR-let-7a and anticancer drug (doxorubicin) in breast cancer. (Reproduced with permission from Yin et al. [55]). **B** Magnetic nanoparticle-based microRNA and hyperthermia therapy to enhance the treatment brain cancer. **a** First, functionalized MNPs are delivered to glioblastoma cells under magnetic field to release miRNA let-7a and induce hyperthermia followed by cellular apoptosis. **b** Structure and designing of functionalized MNPs with let-7a miRNA with polyethyleneimine (PEI). (Reproduced with permission from Yin et al. [56]). **C** Synthesis of miR-198 antisense functionalized magnetic for liver cancer: illustrated steps, (i) functionalization of MNP, (ii) cellular uptake; (iii) selective capturing of miR-198 post (iv) cellular lysis, separation, (v) quantification of miR-198. (Reproduced with permission from Gessner et al. [57])

Often in research applications, magnetic particles are utilized only as carriers to capture the analytes that are trapped on the surface/eluted from magnetic particles by high salt washes and column-based extraction. For instance, the use of super-paramagnetized streptavidin beads to capture biotinylated proteins or nucleic acid in tissue or cellular lysates [11, 17, 44] or, a functionalized MNP to serve as a “bio” label for detection applications [39–41, 44]. There is increasing interest in magnetic nanoparticles (MNPs) for a wide variety of biomedical applications including their functionality in small RNA therapeutics, diagnosis and treatment of cancer, infectious diseases, viral delivery, as well as in vivo imaging for various complex diseases (Fig. 4). Given the current timeline, MNPs are primarily used in biomedical applications such as drug delivery, in vivo imaging, genetic manipulation, immunoassays and cell sorting, *to name a few* [45]. Given such rapid progress in the field and our deep interests in specific disease models, we provide a compilation of functionalized MNPs with their proof-of-concept applications, and respective clinical trial identifiers with the most relevance in Table 1.

**Table 1:** Table summarizing the recent applications of functionalized MNPs in various biomedical applications with respective clinical trial identifiers, as appropriate

Magnetic nanoparticle	Functionalization	Application	Applicable model systems		Clinical trial ID	Ref#
			In vitro	In vivo		
Feredix	Dextran-coated SPION	Liver, spleen, bone marrow imaging			Discontinued (toxicity)	[30]
Magnablate	Iron nanoparticles	Thermal ablation		Prostate cancer	NCT02033447	[136]
Feraheme/ferumoxytol	Iron oxide-carboxymethyl dextran	Monitor response to bevacizumab therapy		Glioma	NCT00769093	[124, 135]
	Iron oxide-carboxymethyl dextran	Localize lymph node metastases		Pancreatic cancer	NCT00920023	[124, 135]
	Iron oxide-carboxymethyl dextran	Detect recent myocardial infarction		Myocardial infarction	NCT01995799	[124, 135]
	Iron oxide-carboxymethyl dextran	Vascular imaging		Migraine	NCT02549898	[124, 135]
	VEGF-165 peptide coupling	Cardiovascular imaging/VEGF delivery	HUVEC			[85]
	Iron-oxide-semi-synthetic carbohydrate shell	Iron deficiency anemia, chronic kidney disease		*i.v* injection in patients	NCT00233597	[126]
Ferumoxide	Iron oxide-dextran	Labeling of inflammed cells		MRI imaging of patient forearm	NCT01169935	[88]
FIONs	PEG-phospholipid	Pancreatic islet graft imaging		Rat liver		[173]
Ferrotran® (Ferumoxtran-10)	Iron oxide-dextran	Prostrate cancer		Lymph node imaging	NCT04261777	[174]
(Gal-PEI-SPIO)	Galactose (Gal) and polyethylenimine (PEI)-modified MNP	siRNA duplexes targeting *c-Met*	Hepa1–6 cells	Hepatic tumor model in C57BL/6 mice		[175]
(ZnFe_2_O_4_-mSi)core nanoparticle	Magnetic zinc-doped iron oxide with mesoporous silica shell	let-7a microRNA + doxorubicin payload	Hela cells	Xenografted nude mice		[21]
Magnetic nanoparticle formulation (MNPF)	beta-Cyclodextrin and PEI coated iron oxide core	miR-145 delivery	HPAF-II, AsPC-1			[176]
MPEI-PEG-magnetic nanoparticles	PEI and PEG coated iron oxide core	miR-205 payload delivery	C4–2, PC-3 cells			[177]
Fe_3_O_4_-SPION	Folic acid (FA) + disulfide-(PEG)-conjugated (PEI) complexed	MRI, siRNA delivery in gastric cancer	SGC-7901			[178]
SPION	T40 dextran coated, epichlorohydrine stabilized	MRI of lymph node, liver, intestine		Pig model		[179]
PEI nanoparticle	Fe_3_O_4_-PEG-LAC-chitosan functionalized	Survivin-siRNA targeted delivery	MCF-7, K562 cells			[147, 180]
SPION	Gemcitabine (Gem)-loaded PLGA-PEG functionalized	Targeted drug delivery	MCF-7			[21]
Iron oxide NP	Chitosan coated, gemcitabine (Gem)-loaded	Targeted drug delivery	SKBR and MCF-7			[181]
SPIONs	Daunomycin-loaded	Targeted drug delivery	HeLa			[182]
	PSMA targeted docetaxel-loaded	Targeted delivery	PC-3			[127]
Ferumoxide	Poly-l-lysine coated	Magnetic targeting in stroke	HB1.F3	Rat		[183]
Paramagnetic perfluorocarbon nanoparticle	Fumagillin loaded, rapamycin loaded, PEG-PEI-gadolinium: ETPA conjugated	αvβ3-integrin-targeted systemic delivery		Rabbit		[184, 185]
	Rapamycin loaded, PEG-PEI-gadolinium: ETPA conjugated	avb3-targeted local catheter delivery		Rabbit		[185]
IONPs	Polyacrylic acid-*co*-maleic acid (PAM) coated	tPA delivery	HUVEC	Rats, human blood		[186–188]
	Amine PEG coated with BSA/ATF protein surface conjugation	Urokinase delivery	PANC02	Mice		[189]
	Streptavidin-coated	miRNA-141 detection		Human serum		[42, 190]
	Streptavidin coated biotin labeled	miR-21 detection	LC–ESI–MS-MS-based	MCF-7		[191]
	EDT coated and DOX loaded	miR-155	bEnd.3, MDCK-MDR, U251			[130]
	Streptavidin-coated and HepB Ab conjugated	Electrochemical detection of HepB	Sandwich ELISA-based			[192]
	Streptavidin-coated and biotinylated HIV-DNA probe conjugate	Viral detection	Electrical impedence based			[193]
	Streptavidin-coated	miRNA detection (let-7b)	SEM, DLS based characterization			[194]
Fe_3_O_4_-virus-magnetic-MIPs (virus-MMIPs)	Green self-polymerization strategy using dopmaine imprinting	Hep A virus detection	CHO cells	Human serum sample		[195]
Fe_3_O_4_ NPs	PEI coated	Genome editing by CRISPR/Cas9	HEK-293 cells			[196]
Superparamagnetic particles	Tosyl group and Influenza protein coated	Viral detection by immunomagnetic assay		Saliva sample		[197]
Graphene oxide MNP	Carboxyl group	miR-122	Fluorescein-labeled HRP-CRET			[198]
Zinc ferrite (ZnFe)	Silica coated and amino (–NH_2_) modified with carboxylic polymers	SARS-CoV-2 RNA capture	Automated in vitro RNA extraction			[156]
BNF-80	Coated with protein A and SARS-CoV-2 spike protein antibody	SARS-CoV-2 (virus) detection	Spectroscopy-based			[10]
Fe_3_O_4_ NPs	Amino (–NH_2_) modified and poly amino coated to generate poly-NH_2_-MNPs	SARS-CoV-2 RNA capture and detection	Spectroscopy and qPCR-based	Nasopharyngeal swab samples		[157]

### Magnetic nanoparticle-based RNA sensors and technologies

RNA interference (RNAi) or silencing is a post-transcriptional phenomenon in eukaryotic cells to silence target messenger RNA/s [46]. In the mammalian system, endogenous gene silencing is predominantly orchestrated by small non-coding RNAs [microRNA, miRNA and/small interfering RNA (siRNA)], which could be leveraged to target genes (mature messenger RNA) in the mammalian system [46, 47]. This principle is exploited to perform loss-of-function (knockout or, knockdown) studies, which harnesses the potential for the development of gene silencing-based therapeutic opportunities [48]. In 2018, the first siRNA drug Onpattro (*aka* Patisiran), which targets transthyretin (*Ttr*) gene for the treatment of peripheral nerve disease in adults was approved by the U.S. Food and Drug Administration (FDA). This remarkable progress, in combination with the identification of the newer and novel disease-related target genes, has catalyzed several innovative research and funding opportunities to design and discover novel siRNA drugs. Despite this promise, current methods for effective siRNA/miRNA drug delivery still encounter major roadblocks in pre-clinical and clinical investigations causing early stage failures [49, 50]. Existing siRNA delivery strategies mainly include lipid or polymer-based siRNA/miRNA delivery systems that present charge discrepancies and high molecular weight due to which non-viral oligonucleotide-based therapeutic treatments have emerged [49, 51–54].

Integrating magnetic nanoparticle-based delivery presents much higher efficiency due to simpler surface modifications, higher surface area and material biocompatibility [58–62]. Therefore, MNPs can be functionalized by various means with peptides or, oligonucleotides to promote cellular uptake and internalization for plasmid transfection, and/or target gene silencing (Fig. 3A, B) [63]. For example, polyethyleneimine (PEI)-coated MNPs with ferric oxide core have resulted in high efficiency RNA delivery to impair tumor cells, delivery of siRNAs targeting B-cell lymphoma-2 (*Bcl2*) and Baculoviral IAP repeat-containing 5 (*Birc5*) genes in cancer cells with efficacious outcomes [64].

MicroRNAs (miRNAs) are 18–21 nucleotides long, single stranded non-coding RNAs with pleiotropic functions that are products of RNA Polymerase II driven transcription which target mature mRNAs in the cytoplasm based on the degree of sequences complementarity (seed, 2–8 nucleotides) between the 5′ end of the miRNA and 3′ region of the target mRNA [41, 65–67]. MiRNA-dependent activity is primarily post-transcriptional that results in either total mRNA degradation or halting of translation machinery, which in both cases represses target gene expression [66, 68]. Emerging pre-clinical and clinical evidence has established that miRNAs are secreted in bodily fluids suggestive of effective early stage biomarkers and therapeutics [69, 70]. Currently employed detection methods such as, miRNA microarray or, miRNA/small RNA sequencing, probe-based in situ hybridization, northern blotting or, quantitative-polymerase chain reaction (qPCR), hybridization-based methods, surface plasmon resonance (SPR) imaging provide information depending on the expression levels of miRNA/s between tissues, body fluids or cellular systems between diseased and healthy controls [70–74]. Nevertheless, this array of high throughput and sensitive methods encounter numerous technical limitations including, expensive reagents, linearity of quantification, detection limit and sensitivity, reproducibility and precision, matrix effect from sample types (water vs. plasma vs. media supernatant), half-life and stability of miRNAs and detection reagents, normalization criteria with relevant reference genes given their expression in cell/tissue types [57, 70, 75–77]. Therefore, the aim of selectively sensing, enriching and capturing the changes in miRNA expression profile in a wide range of sample types is critical for the diagnostic accuracy of functionalized MNPs to provide enhanced therapeutic features when compared to traditional approaches.

As a possible solution, enriching the absolute levels of miRNAs in biological samples through the use of magnetic extraction or magnetic field can be deemed as a powerful approach for a more robust and reliable readout in healthy versus diseased states. Therefore, the type (i.e., functionalization features) and size of the MNP are of key importance in the context of achieving efficient extraction and biosensing. In the past decade, several types of magnetic nanoparticles have been efficiently used in sensing of numerous miRNAs in biological systems [42, 74]. For instance, miR-21 has been detected using assemblies of nano- (Fe_2_O_3_) particles in combination with Pt nanoparticles, and polydopamine–functionalized (Fe_3_O_4_) core nanoparticles with carbon dots have been used for sensing of miR-167 whereas, (Fe_3_O_4_) nanoparticles with carboxyl-modifications have been used in microfluidic concentrator devices to quantify miRNA-200a-3p [74]. Moreover, Fe_3_O_4_-Ag core shell nanoparticles have been employed for capture of miRNAs such as miRNA let-7b in the lower femto-Molar (fM) range presenting an enhanced detection sensitivity. Similarly, graphene oxide-loaded SPIONs have been employed for the detection of cancer-related microRNAs such as, miR-21 and miR-122 sensing in the lower femtomolar to picomolar range [74]. Recently, a zinc-doped iron oxide (ZnFe_2_O_4_) core and a biocompatible silica shell-based functionalized MNP was developed for the simultaneous delivery of miR-let-7a in combination with anti-cancer drug doxorubicin to overcome chemoresistance in breast cancer (Fig. 4A) [55, 78]. Similarly, functionalized MNP-based miR-let7a enhance hyperthermia to kill cancer cells in the glioblastoma cell model including the recent development of miR-198 antisense oligo (ASO) captured on surface functionalized on iron oxide core-silica shell MNP for the therapeutic targeting of liver cancer cells (Fig. 4B, C) [34, 57]. It is important to consider that such proof-of-concept studies have been performed in a relatively homogeneous system without the interference of blood, cellular factors, complex tissue architecture and etc., and the number of studies performed in natural body fluids is highly limited. The ability of concentrating circulating miRNAs on the MNPs through specific surface conjugation or functionalization approaches has served resulted in a number of highly efficient miRNA sensing devices [74, 79]. Nevertheless, capturing early changes in miRNA expression in patient-derived biological samples is one of the key features for development of valuable point-of-care testing. Based on current research trends, the future points towards development of cost efficient functionalized MNPs with improved biocompatibility for early capture and detection of circulating miRNAs in the diagnosis of various types of disease [74, 79] which includes sickness related to different types of organ system.

## Functionalized magnetic nanoparticles for vascular diseases

**Fig. 5:**
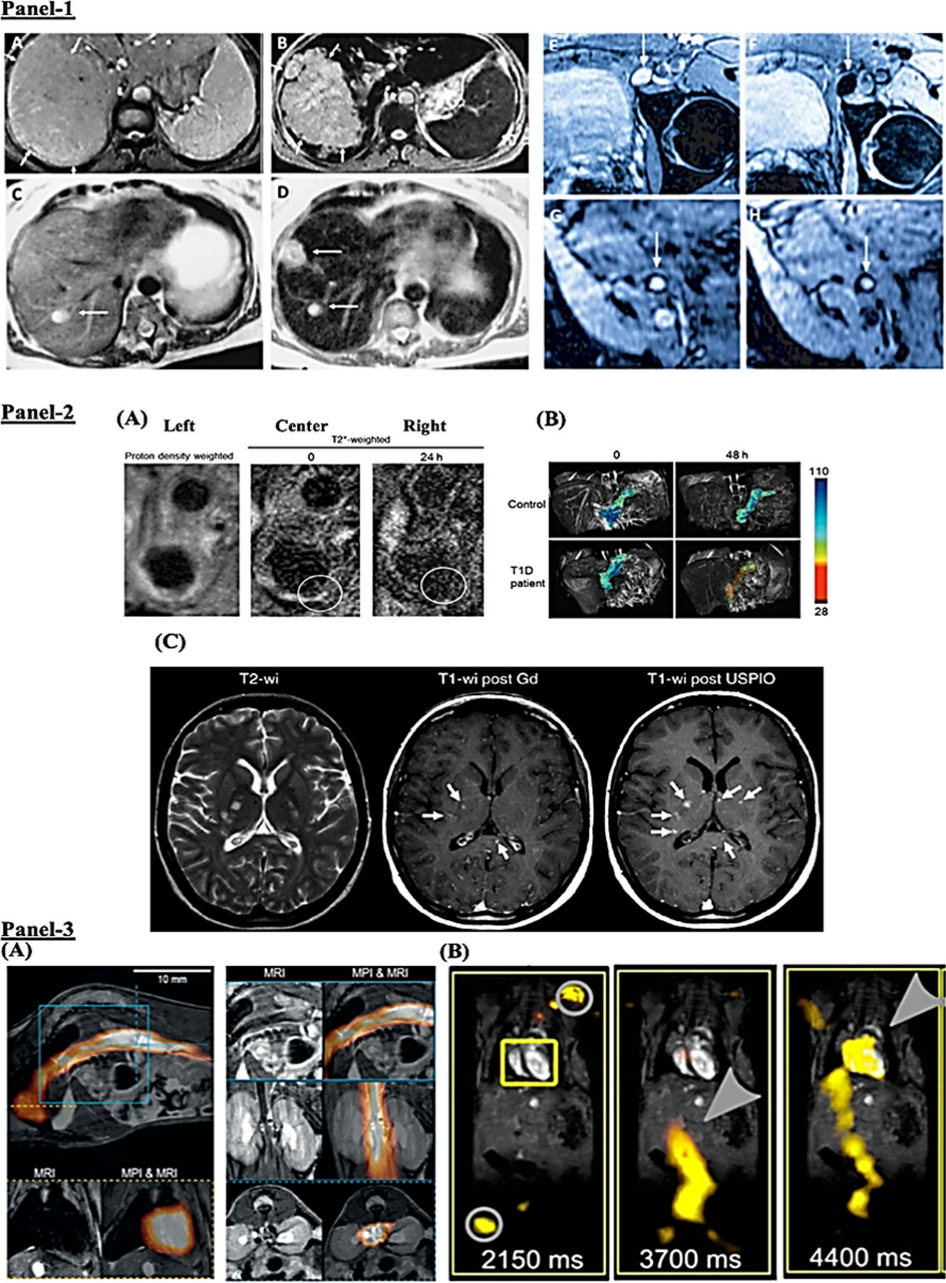
Functionalized MNPs (SPION, USPIO)-based anatomical imaging of human diseases. **Panel 1**: Liver imaging. **A–D** Weighted magnetic resonance imaging of liver hepatocellular carcinoma demarcated with arrows in healthy (**A**) and in disease (**B**) standard imaging (**C**) vs. functionalized SPION-based enhanced (**D**) imaging of liver metastasis (marked by pointed arrows) in a patient with colorectal cancer. Lymph node imaging. **E**–**H** Imaging of lymph nodes in left iliac region in metastatic infiltration before (**E**, **G**) and after (**F**, **H**) ferumoxtran administration. **G** Indicates high UPIO macrophage uptake with arrowheads pointing at no metastasis whereas, **H** presents lack of drug trafficking with persistent metastasis (arrow head). **Panel 2**: Imaging Inflammation with USPIOs. **A** Imaging of external, internal carotid artery (ECA, ICA) in atherosclerosis. T2*-weighted MR images prior (center) and after 24 h (right) of administration (*i.v.*) of functionalized USPIONs. Decreased signal (circled) around the vessel wall at 24 h. **B** USPIO-based pancreatic imagining in diabetes. MR images showing SPION accumulation in a Type 1 diabetic (T1D) patient *vs.* healthy individual. Alterations in the pancreatic microvasculature due to insulitis cause leakage of USPIO particles that can be detected in the inflamed tissue by magnetic resonance imaging. **C** Contrast differences between nanoparticles (functionalized USPION vs. and gadolinium-based) in MRI imaging in multiple sclerosis (MS). Left, multiple hyperintense lesions in the non-contrast-enhanced T2-weighted image. Center, gadolinium-based imaging showing lesions (three arrows). Right, functionalized USPION-based imaging showing six lesions (three additional arrows), highlighting added value for disease diagnosis. **Panel 3**: MR imaging with SPION magnetic particles. Functionalized circulating SPION (ferucarbotran-based) visualized with anatomical information. **A** Image showing SPION circulating through the heart. The top and bottom right images indicate presence in inferior vena cava (sagittal, coronal and transverse orientation). **B** In vivo measurement from a beating mouse heart by MRI and overlaid with traveling wave MPI data. No signal detected at 2150 m post *i.v.* injection of ferucarbotran-based SPION (yellow box), whereas, grey circle indicates the signal of the marker points. Presence of SPIONS in the artery a leading and targeting to the heart 3700 ms, and at 4400 ms. (Reproduced and adapted with permission from Dadfar et al. [91])

Vascular diseases are leading cause of adult disability and mortality worldwide. Alterations/defects in arteries, veins and capillaries are one of the underlying features of vascular diseases, which result in atherosclerosis, cardiovascular diseases, stroke, aneurysm, blood vessel inflammation (vasculitis), deep vein thrombosis [80]. With regards to therapeutic interventions, non-invasive (drug) based targeting are employed in the acute phase to reduce underlying risk factors such as altered cholesterol or blood pressure however, the limited half-life, poor specificity of drugs in the circulation as well as in the vasculature limits the efficacy of current therapeutics. On the other hand, for chronic diseased states, invasive procedures such as angioplasty, surgery-based removal of atherosclerotic plaques or stent implantation are *go-to* strategies. However, these interventions damage the vascular wall, causing restenosis or thrombosis therefore, implying the urgent need for novel treatment and therapeutic options [81–84]. Existing therapeutic strategies involve surgical intervention along with administration of local anti-proliferative or, anti-inflammatory agents [84]. However, these methods are confounded by undesirable side effects and pharmacological limitations including drug absorption, altered blood flow at surgical site, lesions, high drug elimination rate, anatomy-induced vascular bifurcations during surgery. Therefore, several reports have employed a combination of MNPs and magnetic field strength to improve the half-life and site specificity of administered drugs. For example, the use of super-paramagnetic iron oxide nanoparticles (SPIONs) and ultra-small superparamagnetic iron oxide (USPIONs) nanoparticles as MRI contrast agents due to their stability in circulating blood and improved uptake profile in heart, pancreas, liver, spleen and other organs [85, 86] (e.g., Fig. 5, Panel 2A, B, Panel 3). In fact, the SPION-AMI-25, prepared from soluble magnetite (Fe_3_O_4_) was one of the very first magnetic nanoparticles used for blood vessel imaging, with a lower capillary permeability over regular USPIO nanoparticles. In a disease such as atherosclerosis, efficient imaging and visualization of plaques are critical for diagnosis and treatment. Proof-of-concept examples for application of functionalized MNPs include the use fluorescently labeled SPION nanoparticles, PEG (poly ethylene glycol)-conjugated nanoparticles, polymer-coated SPIONs, gold coated SPIONs, high-density lipoprotein-like magnetic nanostructures for magnetic targeting of cellular systems [87, 88]. Recently, administration of drug (paclitaxel)-loaded MNPs or, MNPs loaded with the anti-angiogenic drug fumagillin as well as rapamycin-loaded MNPs when administered under the external magnetic strength have resulted in improved drug pharmacokinetics (such as deposition/absorption, lower elimination rate), causing reduced in-stent restenosis and inhibition of plaque angiogenesis [83, 89, 90].

Therapeutic interventions for human diseases must be long lasting. Gene therapy approaches are deemed efficacious over small molecule based or protein-based therapeutics. In this approach, both viral/non-viral delivery vectors have been tested, with viral-based strategy resulting in the most promising outcomes given the stability in gene expression changes [92–94]. However, the administration of viral-based gene delivery may disrupt the blood flow triggering hypoxia/ischemia and endothelial activation. A handful of recent studies have aimed at functionalized MNP-based gene transfer within the active vasculature. Functionalized adenovirus-loaded MNPs have been reported as efficient gene delivery system in the in vivo vascular network in a rat model of stent angioplasty [93–95]. Moreover, lentiviral-based MNP complexes have been tested under external magnetic field to improve local transduction of native mouse aortic endothelium whereas, utilizing external magnets on the abdominal wall was shown to cause altered tissue distribution after intravascular injection of the functionalized MNP complexes in vivo [95, 96]. Nevertheless, these investigative reports are limited due to the inherent complexity of biological systems and limitations in model systems. Moreover, in atherosclerosis the vascular endothelium undergoes dramatic changes including cellular loss, bleeding and hyper coagulation or, apoptotic/necrotic cell death both as a result of disease as well as during prolonged/extensive surgical procedures. Therefore, cell type specific targeted gene or, cell-based therapies are deemed as a favorable alternate to restore vascular function and to prevent the many post-surgical complications including thrombosis and restenosis. The current strategy is directed to cell population and employs cell replacement using catheters, which restores blood flow however, also causes detachment of cells from the vascular endothelium, which is a technical concern. Hence, restoring the blood flow without altering the vascular bed is critical. Thus, a combination of functionalized MNPs with improved bioavailability profile and magnetic fields may provide better therapeutic outcomes with faster recovery rate in patients. In principle, the strength of the external magnetic field will guide the MNP-loaded drugs and endothelial cells with an improved retention rate, minimal off-site accumulation and specificity at the site of therapeutic intervention (e.g., Fig. 3D). In one study, Lentiviral-based MNP complexes were injected in the blood stream in a murine model to target the MNP-labeled vascular endothelial cells, which were subjected to magnetic field by using a specially designed magnet to overexpress of *eNOS* gene in the labeled endothelial cells to improve the angiogenic factor nitric oxide (NO) production, critical for vascular toning [95].

In spite of these promising applications, it still remains unclear if such experimental strategies can improve therapeutic outcomes in pre-clinical and clinical models. More proof-of-concept studies involving the application of functional genes/proteins for key biomarkers for endothelial dysfunction/activation e.g., *Vegf*; *eNOS*; pro-coagulation markers such as, *SerpineI*; *Thbd* (Thrombomodulin) with an aim to enhance vascular function in early stage vascular diseases is needed. The site-specific delivery of drugs, viral/cell loaded MNPs in the vasculature using a under the influence of optimized magnetic fields can increase the therapeutic index with minimal adverse effects. Recent clinical trials of gene therapy in the vascular systems have led to unsatisfactory results, thereby revealing the need for novel therapeutic concepts and novel functionalization methods [97, 98]. Hence, extensive investigation is needed to improve our knowledge and understanding on the impact of surface modifications and functionalization’s of MNPs for compatibility under blood flow, active vascular system and across various cellular types demands extensive improvement and clarity [92]. In summary, the combination of genetic and cellular approaches together in combination with functionalized MNP’s will open up new avenues for effective and targeted vascular therapies.

## Functionalized magnetic nanoparticles in the neurological system

Neurodegenerative/neurological diseases are a leading cause of adult disability and second leading cause of global death. In the past few decades, the absolute number for neurological disease-associated deaths (~ 30%) and disabilities (~ 15%) have significantly skyrocketed, especially in the developing nations, which is anticipated to worsen in the coming years as a consequence of population growth and ageing [96, 99]. The primary treatment or intervention strategies are based on pharmacological or surgical methods. However, the past few decades have witnessed increased application of novel neuro-modulatory approaches such as the deep-brain stimulation (DBS) therapy which is approved for Parkinson’s disease that employs ultra sound waves, optogenetics to control neural activity, as well as transcranial magnetic stimulation of human motor cortex, electroconvulsive therapy for depression and psychiatric illness, *to name a few* [100–103]. Magnetic fields can traverse through the body without deleterious effects and without changing the anatomy thereby suggestive of their utility in the delivery of stimuli/signals to deeper targets in the human body. Modulation of a given neural circuit/s in the deep brain regions is one of the leading aims of CNS therapies. Magnetic-based neuronal stimulation include techniques such as transcranial focused ultrasound [102], temporally interfering high-frequency electric fields [104] infrared light illumination [105], and near-infrared coupled to plasmonic nanoparticles for heat-dependent neuromodulation [106], nanoparticle-based transcranial optogenetic stimulation of deep-brain structures [106]. Surprisingly, such methods are limited in their resolution and penetration depth when compared to magnetic nanoparticles. MNPs in a uniform magnetic field have promising laboratory results in the context of neural stimulation, activation, regeneration and restoration of neural circuits [107]. Till date, several minimally invasive neural stimulation strategies have been employed. For instance, several studies demonstrating the stimulation of neural regions following injections of fabricated MNPs in combination with viral delivery, DNA or peptide delivery have been elegantly discussed in recent reports [108].

### Bypassing the blood brain barrier (BBB)

**Fig. 6:**
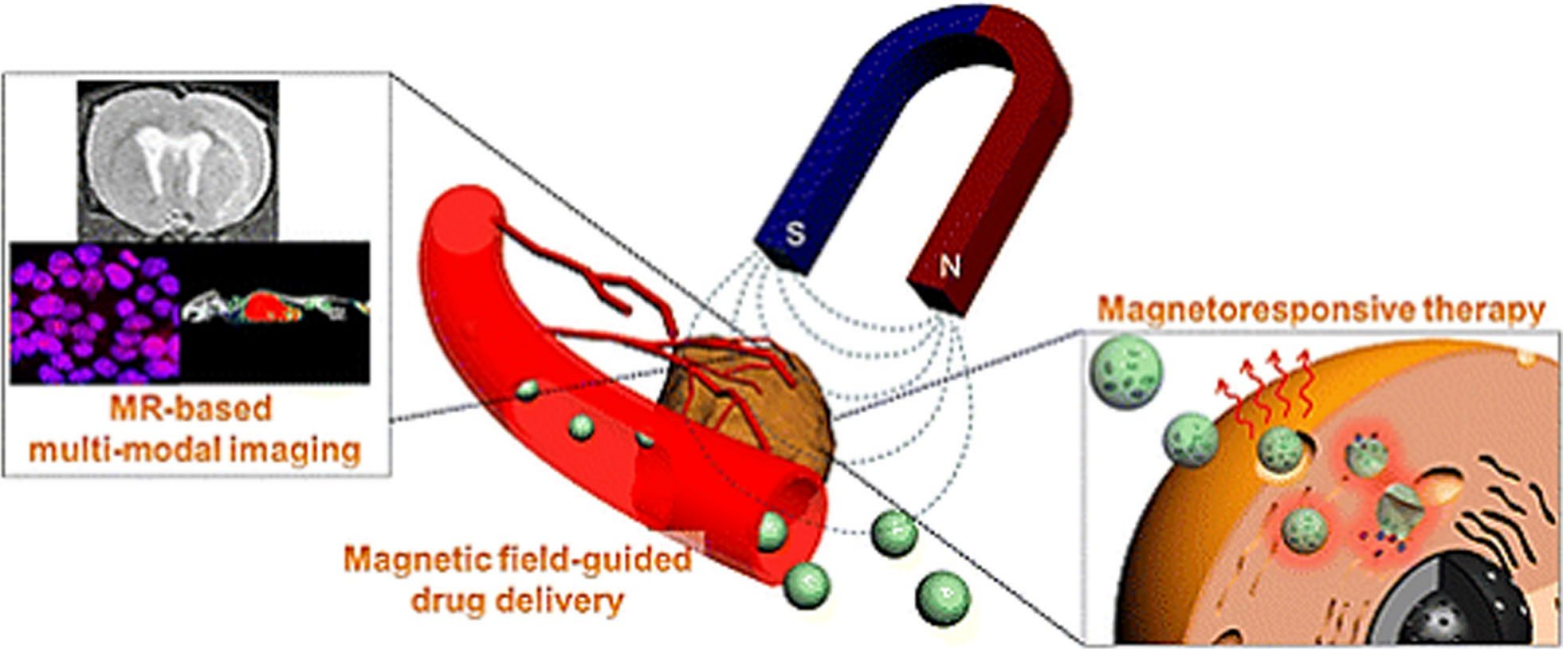
Magnetic field guided drug delivery via the vasculature and multimodal MRI-based imaging using functionalized MNPs. (Reproduced with permission from Lee et al. [16])

Importantly, pathological conditions of the central nervous system (CNS), including stroke, hemorrhage and brain tumors, present a surmounting challenge for imaging and therapeutic delivery due to the selective properties of the blood brain barrier (BBB). The selective barrier properties of the BBB prevent drugs from reaching the brain parenchyma thus impairing the therapeutic bioavailability, which has been one of the major reasons underlying failure of clinical trials for neurodegenerative diseases [109, 110]. The BBB is formed by vascular endothelial cells (ECs) lining the capillary wall connected via tight junctions, surrounded by astrocyte end-feet, and pericytes that embedded in the capillary basement membrane [111]. The BBB functions to maintain brain homeostasis and prevents the intrusion of potentially damaging molecules into the brain parenchyma. The molecular selectivity of the BBB ensures passage and transport of specific molecules (e.g. glucose, water, amino acids) by passive diffusion that is critical for neurovascular homeostasis. Tumors, including gliomas, are inaccessible to chemotherapy, largely due to barrier selectivity. Several experimental approaches to bypass the barrier have been attempted for the delivery of chemotherapeutics, such as the usage of lipid mediated transport, intrathecal drug administration; barrier disruption by osmosis, or biochemically by vasoactive substances [112], or via localized exposure to ultrasound waves [113]. Additional methods are focused on molecular approaches such as, the use of endogenous transporters, Transcytosis (receptor mediated), and blocking of active transporters (e.g., *p*-glycoprotein) or via regulation of trans-endothelial migration. Interestingly, studies have found that vectors targeting BBB transporters, such as the transferrin receptor (TfR) remain entrapped in the brain’s vascular endothelial cells or capillaries and fail to reach the parenchyma [114, 115]. On the other hand, surgical/physical methods for drug delivery involve intracerebral implantation and convection-enhanced distribution. These existing mechanisms have proven less successful against malignant gliomas, which are aggressive with high lethality and high relapse rate with a median survival of ~ 15 months in adults post diagnosis [116–118]. Such challenges have led to growing interest in ferrous-based magnetic materials for overcoming BBB’s selective properties and improve magnetic-based imaging and targeting [112, 119–125]. Under external magnetic field magnetite, (Fe_3_O_4_)-core and maghemite, (γ-Fe_2_O_3_)-based MNPs function as excellent platforms for organ specific targeting and imaging, such as magnetic resonance (MR) or MR-based multimodal particle imaging (MPI) (Fig. 6).

In this context, iron oxide nanoparticles (IONPs)-based and USPIO-based systems have been extensively studied for of the identification of lesions, tumors with an improved imaging, drug loading and release profile (Fig. 5, Panel 2C). For instance, the FDA approved drug ferumoxytol (brand name: Feraheme) containing MNP with iron oxide core is prescribed for the anemia, is also used as the core for nanocomposites of iron oxide nanoparticles (IONPs) to modulate immune cell polarization in tumor model’s indicative of its functional versatility in multiple disease models [112, 126, 127]. One of the key advantages of using IONPs is that magnetic-based methods can be used to alter and facilitate their movement cross the BBB via traversing through the endothelial junctions [86, 120, 128, 129]. In a recent study in rat model, SPIONs functionalized with polyethylene glycol (PEG), polyethylene imine (PEI), and Tween 80 exhibited effective passage across the BBB under an external magnetic field, with improved MNP localization in the cortical regions of the brain [114]. In other studies, iron oxide core MNPs were functionalized by starch coating and tested to target brain tumor using in vivo model of glioma where magnetic targeting improved the therapeutic profile of functionalized MNPs in tumor site with efficient passage through the BBB [129]. In another study, drug and gene-loaded magnetic liposomes were investigated to treat rat glioblastoma [130]. Such multifunctional approaches of functionalized MNPs are of particular interest given the improved and efficacious outcomes in initial investigations.

Functionalized MNPs are also under active investigation for anti-HIV therapy. Brain is often claimed as of the main sources of peripheral virus (HIV) production, which is exacerbated under the influence of external mediators including drugs of abuse, immune dysfunction and co-infection [131]. The low delivery rate of current anti-retroviral drugs (ARTs) across the BBB prevents the targeting of viral lifecycle in the brain cells. The use of magnetic nanocarriers has been postulated to target latent virions in the brain, as well as for the treatment of neurological sequelae caused by HIV damage to the CNS (HAND, *aka* HIV-associated neurological disorders) such as, neurocognitive impairment and HIV-associated dementia (HAD). In general, nanocarriers have been used for delivering anti-HIV drugs to suppress viral infectivity, mitigate oxidative stress and improve synaptic activity and neuronal function [86, 132–135]. However, the utilization of MNPs for targeted drug delivery across the BBB remains a key challenge to overcome. Till date, no commercial applications of magnetic targeting in human subjects have been performed, and the only truly marketed clinical application of functionalized MNPs consists of IONs that are used as contrast agents for magnetic resonance and imaging. The poor retention leading to impaired drug uptake upon the removal of external magnetic field is a major limitation of the MNP application [112]. In this regard, novel strategies to speed up the internalization and drug retention processes by improving the functionalization of MNPs could be the key to overcome existing technical challenges.

## Application of functionalized MNPs in cancer therapeutics

**Fig. 7:**
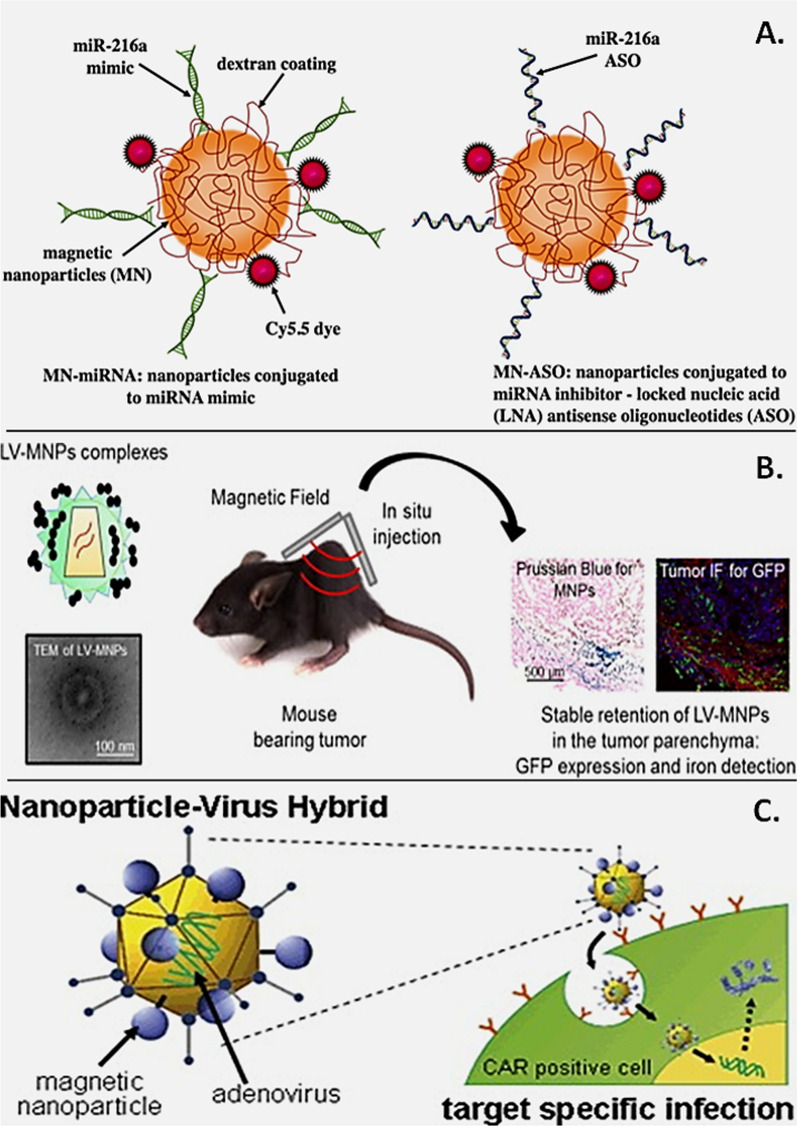
Multi-application features of functionalized MNPs in therapeutics. **A** Schematic of dextran-coated functionalized MNP conjugated with near infrared fluorescent dye (Cy5.5), miR-216a mimic, or inhibitor/anti-sense oligo. (Reproduced with permission from Wang et al. [148]). **B** Functionalized MNPs coated with Lentiviral (LV) vectors used as vehicles for therapeutic targeting in mouse tumor model. Tissue histochemistry results showing stable retention of functionalized LV-MNPs in the tumor tissue. (Reproduced with permission from Borroni et al. [149]). **C** Functionalized hybrid MNPs for Adenoviral machinery for therapeutic targeting of oncogenic cells expressing chimeric antigen receptors (CAR). (Reproduced with permission from Huh et al. [150])

Cancer is a leading cause of worldwide mortality, accounting for nearly 10 million deaths in 2020 [136]. It is a complex disease characterized by cascades of genetic and epigenetic alterations with significant molecular and cellular changes leading to aggressive growth, resistance to apoptosis or cell death. As per a recent estimate, 70% of cancer related mortalities were recorded in low-and-middle-income countries that are projected to surpass beyond 85% with a significant economic burden by 2030 due to the aging population [137]. A key concern of current chemotherapeutics is their inability to distinguish between cancerous and healthy cells, causing the death of both cell types culminating in tissue/organ damage. Hence, numerous research efforts are underway to identify strategies to improve targeted drug delivery to cancerous cells without impacting the healthy cellular environment. Moreover, lack of systemic circulation due to impaired vasculature, drug accumulation, failure in tumor targeting and penetration, disrupted drug uptake by tumor cells are additional hurdles that limit the therapeutic prowess of current drug regimens [84]. In this regard, MNPs are anticipated as a potential solution to overcome this challenge, as they can be manipulated structurally and functionally to improve drug delivery, drug pharmacokinetics and minimize cytotoxicity under a given magnetic field strength [138, 139]. A detailed understanding on the fabrication of functionalized MNPs for cancer and related pathologies can be referred elsewhere, which is beyond the focus of this review [139–141]. Currently, a variety of both functionalized and non-functionalized MNPs are in early stages of pre/clinical development with some formulations recently clinically approved for medical imaging such as bowel imaging (Lumiren, Gastromark); and for liver, lymph node and spleen imaging (Feridex I.V., Ferumoxtran and Endorem) (Fig. 5, Panel 1) [139, 140]. In independent studies, multifunctional functionalized MNPs have been developed and tested for targeted delivery of miRNAs and small molecule therapeutics, in combination to overcome chemoresistance (Fig. 7A) [78]. The use of composite nanoparticles of magnetic iron oxide (Fe_3_O_4_) with functionalization’s such as, β-cyclodextrin cross-linked with ethylenediaminetetraacetic acid (EDTA) and trastuzumab incorporated within magnetic iron oxide core nanoparticles have been extensively validated using in vitro models of cancer with promising outcomes in cellular uptake, targeted delivery, reduced cancer cell proliferation and viability, nuclear fragmentations and cancer cell apoptosis [84]. Similarly, functionalized SPIONs consisting of an inner magnetic core (i.e., magnetite, Fe_3_O_4_, Ni, Co; maghemite, γ-Fe_2_O_3_) functionalized with hydrophilic organic polymers (e.g., polysaccharides, dextran, alginate, PEG, poly vinyl alcohol (PVA) or, by targeting ligands (e.g., avidin, biotin, carboxyl groups)) have been widely used and tested to shield the MNPs from the dynamic cellular/tissue microenvironment and increase therapeutic targeting, bio-distribution [139, 140]. Recent examples include the use of novel magnetic-based targeted in vivo delivery of doxycycline (DOX) in athymic nude rats of high-grade intramedullary spinal cord tumor demonstrating *proof-of-concept* towards an efficient drug delivery approach (Fig. 7B) [142]. In other studies, functionalized SPIONs with fluorescein isothiocyanate (FITC), or encapsulated with quercetin have been tested against human colon cancer as well as hepatocellular carcinoma in vitro whose outcomes suggested safe therapeutic biomedical applications with concomitant reduction in cancer cell proliferation and viability [143, 144]. Similarly, experiments with functionalized SPIONs loaded with chemotherapeutics result in significantly reduced chemo-resistance, improved cellular uptake in cancer cells suggesting remarkable tumor specificity for prostate cancer therapy [145]. The susceptibility of cancer cells to hyperthermia (temperature increases to ~ 43 °C for 30–60 min), which triggers cancer cell apoptosis is well a well-established concept [146, 147]. Exploiting this principle, researchers have developed functionalized SPIONs to kill cancer cells by generating local heat by modulating the magnetic field strength (Fig. 7C). Such ideas have been recently utilized in the development of several pre-clinical mice models that have been reviewed extensively in recent reports [135, 139]. In spite, hyperthermia treatment for cancer therapy still remains controversial. Therefore, functionalized MNPs present a strong clinical tool for therapeutic intervention for reducing patient pain, enhancing their life expectancy towards increasing the success of cancer therapies [135]. In principle, size, chemical composition, shape, and assembly of MNPs can strongly affect the magnetic properties thereby impacting biomedical applications, respectively. Overall it must be noted that surface modifications/functionalization of MNPs can affect key characteristics including, biocompatibility, magnetic strength, drug loading capacity, and size-magnetism ratio for special biomedical applications that has been the bottleneck for clinical translation. Hence, effective and smart surface modifications to enhance functionalization of MNPs with the proper coating material and ligand could facilitate more pre-clinical investigation as well as clinical translation to improve cancer diagnosis and treatment opportunities.

## Proof-of-concept application of functionalized MNPs in COVID-19 diagnosis

**Fig. 8:**
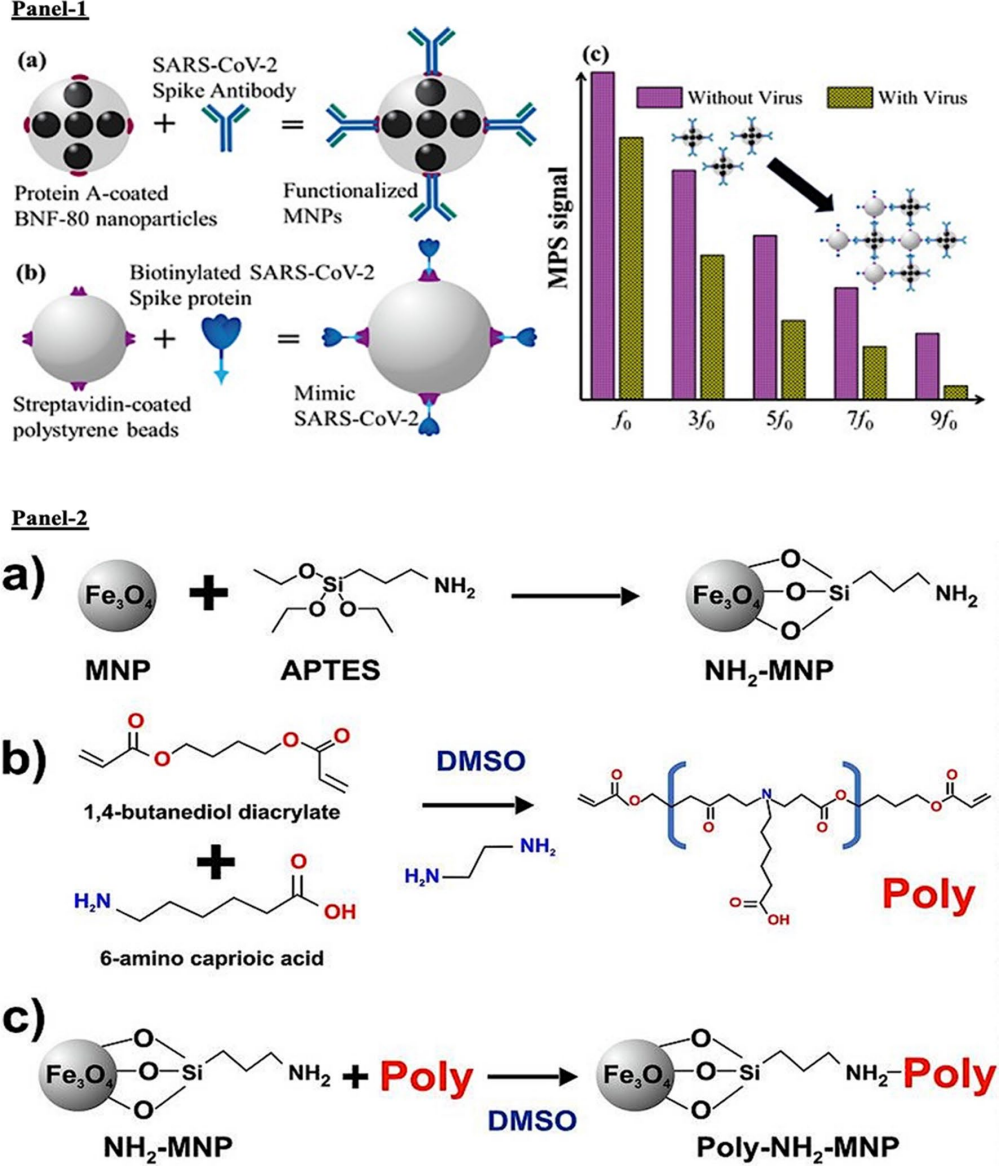
Functionalized MNPs for COVID-19. **Panel 1**: **A**–**C** Designing and fabrication of functionalized MNP for SARS-CoV-2 testing. **A** Schematic of BNF-80 surface modified with protein-A coating and functionalized with viral spike protein antibody to generate functionalized MNP. **B** Schematic of viral mimic generation by conjugation of SARS-CoV2 spike protein on streptavidin-coated polystyrene beads. **C** Representative result indicating signal with and without the presence of virus mimic suggestive of specificity and sensitivity of the MNP. (Reproduced with permission from Zhong et al. [10]). **Panel 2**: **A**–**C** Schematic of the poly (amino)-MNP synthesis. **a** Step 1: reaction of iron-oxide core with aminopropyl triethoxy silane (APTES). **b** Step 2: poly (amino-ester) is synthesized by the combination of 1,4-butanediol diacrylate + 6-aminocaproic acid in DMSO solution via diacrylate-amine polymerization. **c** Step 3: the final (amino-magnetic + poly (amino-ester))-functionalized MNP is represented as poly-(amino) NH_2_-MNP. (Reproduced with permission from Chacón-Torres et al. [157])

The ongoing COVID-19 pandemic caused by the severe acute respiratory syndrome coronavirus 2 (SARS-CoV-2) virus, has caused a global catastrophe with extremely high economic and health burden [151]. Current detection for COVID-19 is primarily relies on techniques including, RT-PCR, chest X-ray, computed tomography (CT) scans, blood-based biomarkers (elevated C-reactive protein, low lymphocyte counts, high interleukins (IL-6/IL-10) [152]). However, these features can be presented in patients in non-COVID patients with underlying conditions including, diabetes, cardiovascular diseases, cancer, *to name a few* implying non-specificities to COVID-19. Therefore, what remains unique is the identification of the spike protein, for instance which the virus uses to engage with host cell ACE2 receptor for cellular entry. Moreover, protein coats of viruses are distinct from the bacteria and other pathogens. Perhaps identifying and targeting of SARS-CoV2 surface proteins may lead to selective, accurate detection of the virus. In an attempt to improve the diagnostic ability during the pandemic, recent works have led towards the development of prototype biosensors such as Fluorescence-based biosensor, electrochemical biosensor (named, eCovSens), optomagnetic sensors for the detection of SARS-CoV2 in clinical samples [153–155]. However, MNP-based biosensing is homogenous that provides a quantitative assessment, measurement of reaction kinetics that can be manipulated based on external magnetic field strength. Therefore, functionalized MNPs are currently under rapid investigation for viral sensing and detection in biological samples. In this regard, recently a simple yet improved MNP assisted RNA-extraction protocol was proposed for extraction and RT-PCR-based diagnosis of COVID-19. In this approach, the core zinc ferrite was functionalized with carboxyl containing polymers that presented improved stability and biocompatibility with a high rate of surface adsorption of viral RNA [156]. In two more recent studies, SARS-CoV-2 spike protein antibody-functionalized MNPs, and poly (amino)-surface modified MNPs were used to detect the viral load in biological samples with increased sensitivity and limit of detection with infectivity profile based on quantitative assessment of the viral load as illustrated in Fig. 8 [10, 157].

Such game changing applications of functionalized MNPs demonstrate the functional versatility in biomedical and theranostic applications which is anticipated to pave the way for development of high efficiency rapid diagnostics not only for SARS-CoV-2, but also for future health pandemics.

## Challenges and opportunities

It is no doubt that functionalized MNPs harbor interesting applications in biomedicine and hence the significant attention in the field of interdisciplinary sciences with rapidly emerging applications ranging from diagnosis to drug delivery and imaging. Intriguingly, the progress is confronted with long-standing conceptual and technical limitations presenting the need for more focus on all fronts.

- Material selection for MNP functionalization: Functionalization is a key step in the designing the net quality, functional efficiency and effectiveness of MNPs. This process involves surface coating of magnetized nanoparticles by polymers including chitosan, dextran, or polyethylene glycol that serve as anchors for peptides, antibodies, and viral particles between the magnetic particle and in the drug for enhanced drug targeting creating an improved hydrophobic and stability profile for the drug in the biological system. Hence, selected materials for functionalization must be assessed for their physical/chemical properties such as electrostatic, hydrophilic/hydrophobic, and affinity interactions since, these properties often lead to uncontrolled drug release.
- Blood flow: The impact of blood flow is a key parameter that ensures effective drug delivery and targeting at the site of injury/lesion. The flow rate in large arteries/vessels is 50–100 times higher than in the capillaries. Hence, appropriate magnetic strength and core particle is essential to ensure the depth of penetration of the magnetic field into the target tissue or anatomical region of the organ system. Impact of blood flow and blood vessel anatomy are additional factors to be considered that influence the strength of the applied magnetic field to guide functionalized MNP-based targeting, which needs further investigations.
- Toxicity: MNPs have been reported to induce toxicity, by activating cells, generation of reactive oxygen species (ROS), accumulation of free metal ions resulting from MNP degradation [31, 158–160]. Therefore, functionalization methods of MNPs must be performed with agents with high stability to limit degradation.
- Biocompatibility: Given the complexity of biological systems, functionalized MNPs must be subjected to rigorous blinded study designs using in vivo and pre-clinical models. For instance, the use of iron oxide or heavy metal core in the MNP design and functionalization has been confronted with questions on clearance, bioavailability, toxicity, biocompatibility *to name a few* [1]. Additionally, how cells and organs respond to these core particles remains unknown. Hence, our current knowledge on biological systems indicates the need for better experimental designs to study the effect/impact of functionalized MNPs using in vivo model systems.
- Particle dispersion: Magnetic field induces attraction within MNPs thereby promoting aggregation in biological systems, which can result in accumulation in tissues and blockage of vessels or capillaries. Therefore, synthesis of mono dispersive MNPs as well as selection of core elements to prevent self-aggregation must be considered while designing of functionalized MNPs [161].
- Quality control measures: Established protocols for quality assurance (purity, sterility, pyrogenic testing) have to be developed to ensure safe use of functionalized MNPs
- Artificial intelligence and Machine learning: Machine learning harbors tremendous potential to accelerate the designing and development of safer functionalized nanomaterial’s for theranostic and healthcare applications. Paucity of high‐quality data and validation models are lacking that present the need for better mathematical models to test the dynamic nature of the biologically relevant systems or compositions. Capturing this complexity is key for generation of robust and predictive AI/ML models. In this realm multifunctional approaches are simultaneously investigated to integrate novel therapeutic and diagnostic agents to amplify the impact of current therapeutic and healthcare modalities. In general, time-dependency, dose-dependent parameters, production costs, efficacy, and patient-specific outcomes are persistent challenges in the development of functionalized MNPs. In particular, the correlation of in vitro and in vivo generated pharmacological datasets could be integrated with in silico models involving Bayesian, support vector machines or deep neural networks to enhance identification and development of effective therapeutic candidates and formulations. Advances in automated synthesis, characterization, high content screening, and predictive modeling of functionalized MNPs in theranostic applications are currently on the rise. Therefore, advances in AI/ML methods on the horizon are anticipated to overcome current roadblocks and facilitate rapid expansion towards designing of safer functionalized MNPs for clinical availability [162–166].

Beyond the scope of this review are the in-depth strategies of functionalization; designing and extensive in vitro biological applications of MNPs. Ultimately, in vivo and pre-clinical investigations are the true testing field to better understand MNPs functionality and applications. To obtain more details and evidence on the designing of functionalized MNPs along with their widespread biomedical applications readers are encouraged to explore many excellent review articles on this subject [12, 21, 43, 91, 107, 147, 167–172]. Overall, the potential for the application of functionalized MNPs is tremendous. Through careful examination of the material properties and improved functionalization features, efficient and groundbreaking outcomes can be obtained for disease diagnostics and drug delivery. We anticipate ideas discussed in this review to open new investigation opportunities and guide communities across biotechnology and biomedical sciences to elevate the field to new possibilities.

In short, one of the biggest in biomedical applications of magnetic nanoparticles lies in dealing with the issue of technology transfer. There are opportunities in this respect for more interdisciplinary approaches, for example, to ensure that the laboratory based experiments can more explicitly emulate the expected conditions that would be encountered in vivo. There is also scope for significant contributions via the mathematical modelling of complex systems, with the objective of understanding more specifically the full gamut of physical phenomena and effects that together determine whether, in the final analysis, a given application will be.

## Data Availability

All the data and materials concerned with the manuscript are available with the corresponding author and can thereby asked.
